# A Review of Platelet-Rich Plasma Use in Patients Taking Non-steroidal Anti-inflammatory Drugs for Guideline Development

**DOI:** 10.7759/cureus.71706

**Published:** 2024-10-17

**Authors:** Tyler Leach, Benjamin Huang, Nicholas Kramer, Shanthan Challa, Richard P Winder

**Affiliations:** 1 College of Osteopathic Medicine, Touro University Nevada, Henderson, USA; 2 Orthopedic Surgery, Valley Hospital Medical Center, Las Vegas, USA; 3 Orthopaedic Surgery, Desert Orthopaedic Center, Las Vegas, USA

**Keywords:** nonsteroidal anti-inflammatory drugs (nsaids), orthopedics, orthopedic sports medicine, platelet-rich plasma (prp), prp injection

## Abstract

Platelet-rich plasma (PRP) is an autologous blood product containing concentrated platelets, growth factors, and anti-inflammatory cytokines that promote healing and regeneration. Platelets release active components through a degranulation process, which is inhibited by certain nonsteroidal anti-inflammatory drugs (NSAIDs). Current deferral guidelines are not established, but NSAIDs are expected to have a time-dose relationship with platelet inhibition. This article aims to review the literature regarding NSAIDs that inhibit a key enzyme in clotting, cyclooxygenase-1 (COX-1), and their effects on bulk platelet aggregation to establish guidelines for deferral times before PRP injections. After searching the literature, 25 studies met the inclusion criteria for this review. Our analysis of the data revealed several significant findings. Naproxen demonstrated inhibition of platelet aggregation lasting at least 24 hours, with possible inhibition even at 48 hours. Studies using indomethacin found recovery by 24 hours, although no other time points were measured. Ibuprofen and diclofenac inhibit platelet aggregation for 6-12 hours, depending on dose. Acetaminophen, a mild inhibitor of COX-1, does not require deferral. These results reinforce the need to defer NSAIDs with COX-1 activity before PRP injections. This review will allow for the creation of deferral guidelines that differentiate NSAIDs based on their length of platelet inhibition.

## Introduction and background

Platelet-rich plasma (PRP) is a centrifuged preparation of autologous blood that contains elevated concentrations of platelets, anti-inflammatory molecules, mediators, cytokines, and growth factors to ultimately improve tissue healing [1,2]. Its preparation can be quite varied as no optimal method has been established, although leukocyte and fibrin content have been included recently on top of fundamental characteristics such as platelet concentration, total platelet count, and concentration of growth factors [3]. In musculoskeletal conditions, PRP injections are often applied in tendinous injury and knee osteoarthritis [1]. PRP has few contraindications as it is derived from a patient’s own blood and thus has negligible risk for immunogenic reaction [4].

**Figure 1:**
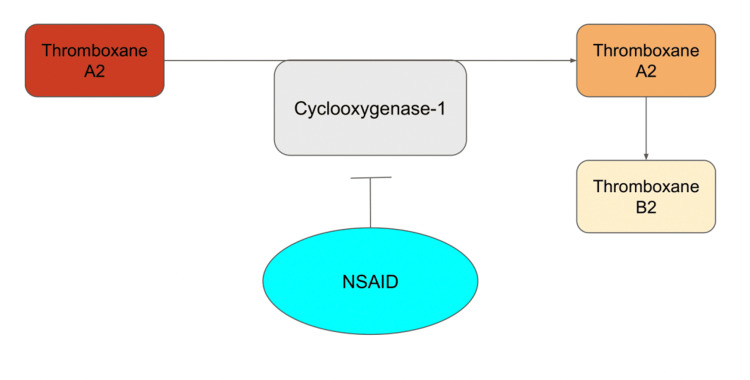
Platelet aggregation pathway. This image is the authors’ original work. NSAID: nonsteroidal anti-inflammatory drug

Nonsteroidal anti-inflammatory drugs (NSAIDs) are a known antiplatelet medication and relative contraindication to PRP injections; they are also a first-line treatment for musculoskeletal conditions [5,6]. PRP releases its cytokines and growth factors in the presence of thromboxane A2 (TxA2) [6]. NSAIDs inhibit platelet aggregation through competitive antagonism of cyclooxygenase (COX) 1 and 2 [7]. These isoenzymes produce inflammatory molecules, including TxA2, which is a necessary component of the biochemical cascade (Figure 1).

NSAIDs inhibit platelet aggregation through antagonism of COX-1. Current evidence suggests COX-2 inhibition does not inhibit platelets and thus is likely acceptable with PRP injections [5]. In contrast, NSAIDs that inhibit COX-1 are deferred aggressively despite a lack of specific guidelines to guide clinical management [8]. The purpose of this review was to categorize the various COX-1-inhibiting NSAIDs based on their respective length of platelet inhibition to provide guidelines for the clinical management of PRP injections in the setting of concomitant NSAID use. The dose of NSAIDs and its relationship to platelet inhibition was also investigated. The medications selected in this review are the most commonly used NSAIDs to treat musculoskeletal conditions.

## Review

Methodology

This systematic review was performed in accordance with the Preferred Reporting Items for Systematic Reviews and Meta-Analyses (PRISMA) statement, along with guidelines for rapid reviews [9,10]. A search was done on PubMed employing the keywords “PRP,” “platelet,” “NSAIDs,” “ibuprofen,” “naproxen,” “diclofenac,” “acetaminophen,” “indomethacin,” and their combinations. The selection process of the studies was done using Covidence (Covidence systematic review software, Veritas Health Innovation, Melbourne, Australia; available at www.covidence.org.). A total of 513 unique papers were screened, resulting in 25 papers included in the final review based on the inclusion and exclusion criteria. Data from the articles were manually extracted, and measures of aggregometry, thromboxane B2 (TxB2) titers, and time since the last dose or time since peak plasma concentrations were compiled. The last search was conducted on August 4, 2024.

Study Selection Criteria

Inclusion criteria included healthy human volunteers, NSAIDs with COX-1 activity (ibuprofen, indomethacin, naproxen, acetaminophen, diclofenac), compared with placebo or “no treatment,” and outcomes were aggregometry or TxB2 titers. Exclusion criteria included studies without a placebo or control group, studies that did not report relevant outcomes, studies conducted among the pediatric population, studies with the wrong route of administration, or retracted studies. The main outcomes, namely, platelet aggregometry and TxB2 titers, reviewed in this article were selected due to their validated measurement of platelet aggregation [11,12]. TxB2 is a stable metabolite of TxA2 and is a quantitative measure of COX-1 inhibition. Aggregometry protocol involves the addition of an activating agent to begin the process of platelet aggregation. This is typically done with varying concentrations of arachidonic acid (AA), adenosine diphosphate (ADP), epinephrine, adrenaline, or collagen. The usage of these activators was documented if a study reported differences in aggregometry due to the concentration or type of activator. Individual studies were tabulated with participant characteristics and the relevant outcomes.

Results

**Figure 2:**
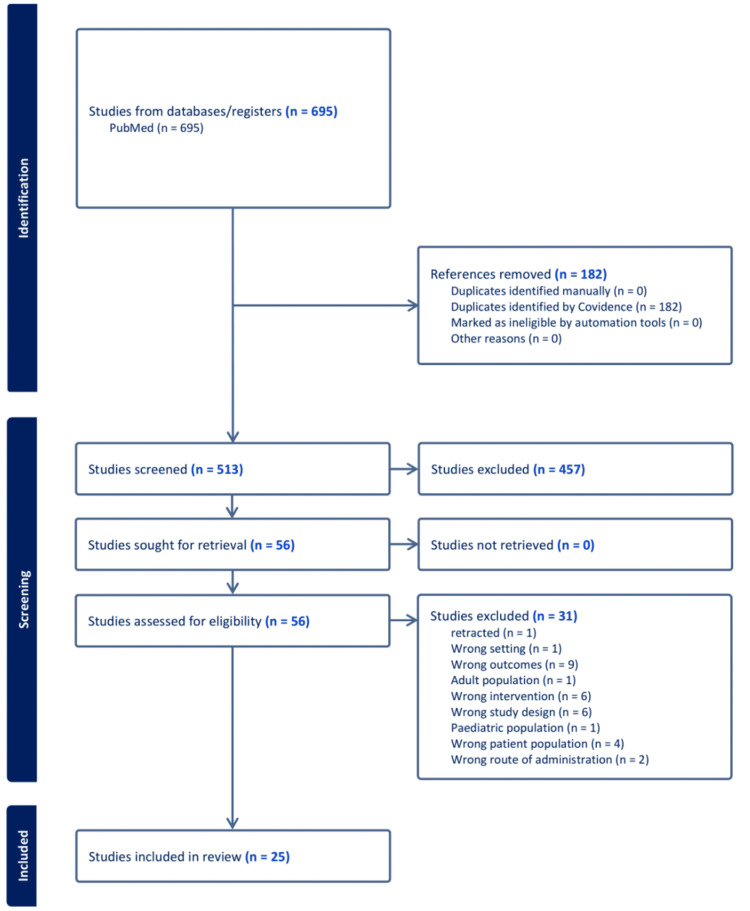
Preferred Reporting Items for Systematic Reviews and Meta-Analysis (PRISMA) flowchart.

A total of 695 studies were identified through the initial search, with 25 studies meeting all the inclusion criteria and totaling 522 participants. Each NSAID was represented in this review somewhat uniformly: eight studies involved diclofenac, five studies involved acetaminophen, six studies involved ibuprofen, five studies involved indomethacin, and six studies involved naproxen. Of the 25 studies that met all the inclusion criteria, 11 were randomized controlled trials (RCTs), 10 were crossover RCTs, and two were in vitro dose-response studies. A description of the manuscript selection process is depicted in Figure 2.

Assessment of Outcomes

Table 1 provides a narrative summary of the studies included in this review.

**Table 1:** Summary of the included studies.

Article title	Author	Journal	Year	Summary
Brief communication: duration of platelet dysfunction after a 7-day course of Ibuprofen	Goldenberg et al. [13]	Annals of Internal Medicine	2005	11 healthy adult volunteers received 600 mg ibuprofen orally every eight hours for seven days. Aggregometry showed increased closure time at 40 minutes, with some recovery at eight hours and some recovery at 24 hours
Assessment of common nonsteroidal anti-inflammatory medications by whole blood aggregometry: a clinical evaluation for the perioperative setting	Scott et al. [14]	World Neurosurgery	2014	12 healthy volunteers received 600 mg ibuprofen three times per day or naproxen 440 mg twice a day for three days. After 24 hours, aggregometry showed no inhibition with ibuprofen while naproxen showed inhibition. At 48 hours, naproxen inhibition recovered in 83% of women and 50% of men. At 72 hours, all platelet function recovered
Dose responses of ibuprofen on platelet aggregation and coagulation in human platelets in vitro	Martini et al. [15]	Military Medicine	2016	Blood samples collected from four healthy humans were dosed to reach 163 µg/mL ibuprofen or higher, reaching two times, four times, eight times, and sixteen times the initial concentration. Aggregometry showed increased closure time at 163 µg/mL using arachidonic acid activation and at two times concentration using collagen activation
The interaction of ibuprofen and diclofenac with aspirin in healthy volunteers	Schuijt et al. [16]	British Journal of Pharmacology	2009	12 healthy volunteers received 50 mg diclofenac three times per day or 800 mg ibuprofen two times per day. After 8 hours, TxB2 titers were reduced by 30% with diclofenac and 83% with ibuprofen
Platelet inhibitory effects of OTC doses of naproxen sodium compared with prescription dose naproxen sodium and low-dose aspirin	Schiff et al. [17]	Current Medical Research and Opinion	2009	48 volunteers received 220 mg two to three times per day versus 550 mg two times per day of naproxen and found similarly reduced (97%+ inhibition) titers of TxB2 compared to 81mg aspirin (98%+ inhibition) 24 hours after the last dose
Effects of celecoxib, a novel cyclooxygenase-2 inhibitor, on platelet function in healthy adults: a randomized, controlled trial	Leese et al. [18]	Journal of Clinical Pharmacology	2000	24 healthy adults took 500 mg of naproxen twice per day. After 24 hours, both platelet aggregation and TxB2 titers were reduced
Valdecoxib does not impair platelet function	Leese et al. [19]	American Journal of Emergency Medicine	2002	62 Healthy volunteers were given either 500 mg naproxen or 75 mg diclofenac. After eight hours, aggregometry showed that patients who took diclofenac had normal platelet aggregation and patients who took naproxen had increased closure times. TxB2 titers were also measured at 8 hours and the diclofenac arm showed recovery while the naproxen arm showed significantly decreased measurements
The impact of selective and non-selective non-steroid anti-inflammatory drugs on secondary hemostasis in healthy volunteers	Schjerning et al. [20]	Thrombosis Research	2009	20 healthy male volunteers received 250 mg naproxen for 21 days. 12 hours after each dose, platelet samples taken from the patients showed inhibition of aggregation
Selective inhibition of COX-2 in humans is associated with less gastrointestinal injury: a comparison of nimesulide and naproxen	Shah et al. [21]	Gut	2001	36 healthy volunteers received 500 mg naproxen two times per day for 14 days. After one to two hours, TxB2 titers were reduced by 98% with partial recovery two days after the last dose. Platelet aggregation was inhibited with AA but less with ADP activation
Effect of non-selective, non-steroidal anti-inflammatory drugs and cyclo-oxygenase-2 selective inhibitors on the PFA-100 closure time	Blaicher et al. [22]	Anaesthesia	2004	44 healthy volunteers received 75 mg diclofenac sodium. Aggregometry showed inhibition at three hours with complete recovery at 12 hours
Comparison of the effect of intravenous ketoprofen, ketorolac and diclofenac on platelet function in volunteers	Niemi et al. [23]	Acta Anesthesiology Scandinavia	1997	10 healthy volunteers were given 1.1 mg/kg diclofenac intravenously. After 2 hours, inhibition was found with adrenaline activation with complete recovery by 24 hours. With ADP activation, no inhibition was found
Propacetamol augments inhibition of platelet function by diclofenac in volunteers	Munsterhjelm et al. [24]	British Journal of Anesthesia	2003	10 healthy male volunteers received diclofenac intravenously to reach 1.1 mg/kg and found increased closure time at 30 minutes with complete recovery after 22–24 hours
Effect of diclofenac sodium (feloran) on platelet aggregation	Tyutyulkova et al. [25]	Methods and Findings in Experimental and Clinical Pharmacology	1984	8 healthy volunteers received 25 mg diclofenac three times per day for eight days. 10 hours after the last dose, 25% inhibition was found
Platelet function following administration of a novel formulation of intravenous diclofenac sodium versus active comparators: a randomized, single dose, crossover study in healthy male volunteers	Bauer et al. [26]	Journal of Clinical Anesthesiology	2010	30 healthy volunteers received diclofenac sodium intravenously (37.5 mg) or diclofenac potassium orally (50 mg). Aggregometry showed inhibition at 1.5 and 3 hours with near-complete recovery after 6 hours for both treatment arms
Indomethacin prevents the long-lasting inhibitory effect of aspirin on human platelet cyclo-oxygenase activity	Livio et al. [27]	Prostaglandins	1982	3 volunteers received 50 mg indomethacin. After two hours, aggregometry showed inhibition, and TxB2 titers were reduced (99% inhibition) with recovery of both at 48 hours
Time-dependent inhibition of platelet cyclo-oxygenase by indomethacin is slowly reversible	Walenga et al. [28]	Prostaglandins	1986	4 healthy adults received 25 mg indomethacin. After six hours, inhibition of TxB2 production was found with recovery by 24 hours
Meloxicam, 15 mg/day, spares platelet function in healthy volunteers	deMeijer et al. [29]	Clinical Pharmacology & Therapeutics	1999	15 healthy volunteers took 25 mg indomethacin three times per day for seven days or 15 mg meloxicam one time per day for seven days. After one hour, aggregometry showed inhibition, and TxB2 titers were reduced (95%+ inhibition).
A comparison of the effects of nabumetone vs meloxicam on serum thromboxane B2 and platelet function in healthy volunteers	vanKraaij [30]	British Journal of Pharmacology	2002	12 healthy volunteers received 50 mg indomethacin. After two hours, aggregometry showed inhibition, and TxB2 titers were reduced
Effects of meloxicam on platelet function in healthy adults: a randomized, double-blind, placebo-controlled trial	Rinder et al. [31]	The Journal of Clinical Pharmacology	2002	82 healthy subjects received 75 mg indomethacin one time per day for eight days. After six hours, aggregometry showed inhibition and TxB2 titers were reduced
Inhibition of human platelet function in vitro and ex vivo by acetaminophen	Lages et al. [32]	Thrombosis Research	1989	5 healthy volunteers received 650 or 1,000 mg acetaminophen. After one hour, inhibition of aggregation was not found while TxB2 titers showed 30–60% inhibition
Influence on platelet aggregation of i.v. parecoxib and acetaminophen in healthy volunteers	Munsterhjelm et al. [33]	British Journal of Anesthesiology	2006	18 healthy volunteers received 1 g of acetaminophen intravenously. After 10 minutes, no inhibition of platelet aggregation nor a reduction of TxB2 titers was found. AA activation at 500 µM showed high variability but possible inhibition
Dose-dependent inhibition of platelet function by acetaminophen in healthy volunteers	Munsterhjelm et al. [34]	Anesthesiology	2005	13 healthy males received acetaminophen intravenously to reach 30 mg/kg. With AA activation, increased closure time was found only at the 10-minute mark with recovering function by 90 minutes. Other activators of platelets found no inhibition
Acetaminophen and meloxicam inhibit platelet aggregation and coagulation in blood samples from humans	Martini [35]	Blood Coagulation and Fibrinolysis	2014	Blood samples from six healthy humans were dosed to 214 µg/mL acetaminophen and found to increase closure time
Point-of-care platelet function tests: detection of platelet inhibition induced by nonopioid analgesic drugs	Scharbert et al. [36]	Blood Coagulation and Fibrinolysis	2007	40 healthy volunteers received 75 mg diclofenac or 1,000 mg acetaminophen intravenously. After 30 minutes, diclofenac showed inhibition while acetaminophen showed no effect

Ibuprofen

Two studies found oral ibuprofen (600 and 800 mg) inhibited platelet aggregation, and after eight hours, one study [13] found some recovery by aggregometry, and the other study [16] found incomplete inhibition of COX-1 activity (83% reduction of TxB2 titers). Two studies [13,14] found that 24 hours was enough time for complete recovery of platelet function by aggregometry. Interestingly, Goldenberg et al. [13] reported that four participants had no response or even increased platelet aggregation after a seven-day course of ibuprofen. Of these four, three were women who were taking oral hormonal contraceptives. For men, 50% showed platelet aggregation, which was recovered by eight hours. Furthermore, Scott et al. [14] reported statistically significant differences (p > 0.01) between men and women aggregation times at 24 hours after a 600 mg oral dose. Martini et al. studied in vitro inhibition using ibuprofen and an arachidonic acid activator. Closure time was increased at 162 µg/mL ibuprofen [15]. This study found that with a collagen activator, a two times greater dose was required to demonstrate inhibition.

Naproxen

Five studies found that 250 mg of oral naproxen inhibited platelet aggregation by aggregometry and TxB2 titers longer than 24 hours [14,17-20]. One study found aggregometry was slowed even 48 hours after administration [21]. Schiff et al. randomized 12 participants to naproxen or aspirin and found no significant difference in platelet aggregometry after 24 hours [17]. Comparing ibuprofen and naproxen, Scott et al. found a difference between men and women 48 hours after oral administration, where aggregation was recovered in 83% of women and 50% of men [14].

Diclofenac

Two studies found that 75 mg of diclofenac after eight hours was enough time for recovery by TxB2 titers [19,25]. Tyutyulkova et al. subjected eight participants to 25mg of diclofenac three times per day and 10 hours after the last dose and found 25% inhibition of platelet aggregation [25]. Munsterhjelm et al. and Niemi et al. titrated patients intravenously to 1.1 mg/kg diclofenac and found no evidence of slowed aggregometry after 24 hours [23,24]. Notably, Niemi et al. noted ADP activation was not inhibited even two hours after intravenous (IV) administration [23]. Blaicher et al. studied the effects of 75mg diclofenac and found closure time was increased at three hours and not significantly increased at 12 hours [22]. In the only study analyzing other formulations of diclofenac, Bauer et al. reported a smaller dose (37.5 mg diclofenac sodium by IV) but complete recovery at six hours using aggregometry [26]. This study also randomized participants to diclofenac potassium orally at 50 mg and found no inhibition at six hours.

Indomethacin

Four studies reported platelet inhibition one to two hours after administration, and one study found recovery at 24 hours after administration [27-30]. One study found inhibition lasted longer than six hours after administration using TxB2 titers [31].

Acetaminophen

Further, 1,000 mg of acetaminophen delivered orally or intravenously was not found to inhibit platelet aggregation by aggregometry [32,33,36]. Munsterhjelm et al. found that 30 mg/kg (70 kg adult = 2,100 mg dose) acetaminophen intravenously did not increase closure times except 10 minutes after administration and using 500uM arachidonic acid activation [34]. The authors’ 2006 follow-up study of one gram of acetaminophen intravenously found no increase in closure time when using 1,000 µM arachidonic acid activation [33]. The authors included data using 500 µM arachidonic acid and found highly variable inhibition with no significant effect. Lages et al. found that 650 mg and 1,000 mg of acetaminophen did not significantly inhibit platelet aggregation, while TxB2 titers showed a 30-60% reduction in concentration [32]. Martini et al. in vitro found 214 µg/mL increased closure time [35].

Table 2 provides a summary of the recommended deferral times and relevant doses.

**Table 2:** Summary of recommended deferral times and the relevant dose. NSAID: nonsteroidal anti-inflammatory drug

NSAID	Relevant dose	Deferral
Diclofenac	25–75 mg	6 and 12 hours, respectively
Ibuprofen	600–800 mg	8 hours
Naproxen	250–550 mg	24–48 hours
Indomethacin	25–75 mg	24 hours or less
Acetaminophen	2,000 mg+	Not necessary

Discussion

Ibuprofen (46 participants) at a 600-800 mg oral dose of ibuprofen reduces TxB2 titers and increases closure times in aggregometry for eight hours [13,16]. Patients taking ibuprofen should defer PRP injections for eight hours or less if a smaller dose is taken. Martini et al. found 163 µg/mL of ibuprofen inhibited in vitro platelet aggregation, but smaller doses were not tested [15]. Pharmacokinetic studies report 40-60 µg/mL peak plasma concentrations after 600 mg oral administration of ibuprofen [37]. NSAIDs are also known to concentrate as much as three times in synovial fluid, which could affect PRP injections indicated for knee osteoarthritis [38]. Furthermore, Scott et al. reported women maintained platelet aggregation after taking NSAIDs and almost a 50% greater aggregation response compared to men after NSAIDs [14]. Women may require significantly shorter deferral times in randomized clinical trials.

Participants taking naproxen (76 participants) demonstrate platelet inhibition for 24-48 hours [14,17-21]. Schiff et al. found no difference between naproxen and aspirin after 24 hours. Naproxen likely has a sustained effect on platelet aggregation [17]; this effect potentially can be explained by naproxen’s elimination half-life of 12-17 hours [39]. As platelet cellular turnover is about 10% per day and 20% of new platelets regenerate platelet aggregation [40], 48-72 hours could prove to be a maximum deferral time for any NSAID.

Participants taking diclofenac (216 participants) show recovery of platelet aggregation after 6-12 hours, depending on the dose (25-75 mg) [19,22,25]. However, future studies could support even shorter deferral times, as diclofenac has a half-life of 1.8 hours [41]. Interestingly, Niemi et al. found that diclofenac did not affect ADP-induced platelet aggregation [23]. Although other reviewed studies using ADP as an agonist generally find weaker inhibition compared to 500 µM arachidonic acid [21,23,33,34], this study’s findings compared to adrenaline could be a direction for future investigation.

Participants taking indomethacin (n = 109) showed inhibition at six hours, with recovery by 24 hours [27-30]. Walenga et al. reported that 25 mg indomethacin completely prevented TxB2 synthesis at two hours and that at six hours, indomethacin assays showed less than 5% remaining concentration of indomethacin in plasma and 50% inhibition of TxB2 titers [28]. In contrast, Rinder et al. found a 95% reduction at six hours but instead used 75 mg oral indomethacin [31]. As the dose is three times larger, it is expected that inhibition is longer lasting. It is clear that indomethacin has a paucity of information to guide deferral guidelines.

Patients taking oral acetaminophen are not likely to require deferral of PRP injections. The studies reviewed (76 participants) show no inhibition of platelet aggregation nor decreases in TxB2 at clinically relevant doses [32-36]. Munsterhjelm et al. found that intravenously, 30 mg/kg (70 kg = 2,100 mg dose) of acetaminophen caused transient inhibition for 10 minutes [34]. While 500 µM arachidonic acid activator showed inhibition transiently, higher concentrations or a different agonist showed no inhibition. The study was repeated with 1,000 mg acetaminophen intravenously with the original 500 µM arachidonic acid method, showing significant variability in the control study, ultimately being unusable for analysis [33]. This series of studies indicates that acetaminophen is a contraindication only at supratherapeutic doses. It also brings into question the validity of studies using 500 µM arachidonic acid activator for increased risk of false-positive results. Furthermore, in vitro analysis showed that 214 µg/mL acetaminophen was required to increase closure time in aggregometry [35], while pharmacokinetic studies suggest 1,000 mg oral acetaminophen reaches a peak plasma concentration of 5.2-7.3 µg/mL [39]. Ultimately, in vitro studies are even less valid than ex vivo evidence available from Munsterhjelm et al. Acetaminophen as adjunct therapy with PRP is a valid, future direction for controlling pain between discontinuing other oral NSAIDs and PRP efficacy to take effect.

To our knowledge, this is the first review that elaborates on the relationship between COX-1 NSAIDs and platelet aggregation. These results provide some guidelines for the currently unestablished deferral guidelines for PRP. Kao et al. [5] established other drug interactions with PRP: COX-2 does not inhibit platelet aggregation and thus is allowed with PRP injections, and aspirin, an irreversible inhibitor of COX-1, definitely does inhibit platelet aggregation. This review addresses a question that the authors suggested: establishing deferral times for COX-1 NSAIDs.

Limitations

The limitations of this review include significant heterogeneity of the studies in the time points measured, agonist used for platelet aggregation, and outcomes measured. Limited conclusions can be drawn as there is only moderate consistency between the times measured and doses used. Different agonists used in aggregometry ultimately created more questions and many studies used 500 µM arachidonic acid and concluded that it is the most sensitive method to determine inhibition of platelet aggregation. Furthermore, one study noted differences in response due to sex [13]. Another study found a statistically significant difference in platelet inhibition between men and women [14]. This could limit this study’s applicability to both sexes. This is another potential direction of study to delineate the effects of PRP between the sexes. Finally, there is also a risk that more relevant articles were missed during the database search as this rapid review did not search multiple databases.

Future directions

Future research could focus on differences in NSAID effects clinically and on laboratory platelet inhibition as Reilly et al. determined the relationship between TxB2 titers and bleeding time [42,43], and eight studies correlated platelet aggregometry with TxB2 titers [18-21,27,29,31-33]. Inevitably, clinical studies are needed to confirm the suggestions of this paper. Kao et al. [5] found no changes in platelet count after oral NSAIDs, but there could be effects from changes in PRP composition, as naproxen has been shown to reduce growth factor concentration for as long as a week [42]. As clinical trials are costly and require longitudinal follow-up, the future direction could be aimed at external validation of TxB2 titers as a measure of platelet function. Reilly et al. initially reported that >96% reduction in TxB2 synthesis was required for a difference in bleeding time [43], and some studies reviewed found no changes in aggregometry with significant reductions in TxB2 synthesis [31,32].

## Conclusions

NSAIDs with strong COX-1 activity require deferral before PRP injections. Naproxen requires 24-48 hours of deferral, likely due to its extended half-life. Ibuprofen and diclofenac no longer inhibited platelet aggregation after 6-12 hours, depending on the dose. Indomethacin had a paucity of data on its platelet activity, but it is clear that it does not inhibit platelet aggregation after 24 hours. Acetaminophen, even at somewhat supratherapeutic doses, does not inhibit platelet aggregation.
